# Importance of preoperative total colonoscopy and endoscopic resection after self-expandable metallic stent placement for obstructive colorectal cancer as a bridge-to-surgery

**DOI:** 10.1186/s12876-023-02888-z

**Published:** 2023-07-24

**Authors:** Shuichi Itonaga, Shohei Hamada, Eikichi Ihara, Hitoshi Honma, Hiroki Fukuya, Akito Ookubo, Taisuke Sasaki, Daisuke Yoshimura, Makoto Nakamuta, Yorinobu Sumida, Naohiko Harada

**Affiliations:** 1 Department of Gastroenterology, Clinical Research Institute, National Hospital Organization Kyushu Medical Center, 1-8-1 Jigyohama, Chuo-ku, Fukuoka, 810-8563 Japan; 2 Department of Medicine and Bioregulatory Sciences, Graduate School of Medical Sciences, Kyushu University, Fukuoka, Japan; 3 Department of Gastroenterology, Kitakyushu Municipal Medical Center, Fukuoka, Japan

**Keywords:** Obstructive colorectal cancer, Bridge-to-surgery, Self-expandable metallic stent, Synchronous cancers, Endoscopic resection

## Abstract

**Background and aim:**

Colonic self-expandable metallic stent (SEMS) placement enables preoperative total colonoscopy (TCS) in patients with obstructive colorectal cancer. Following SEMS placement, it is possible to assess the presence or absence of synchronous proximal colon cancers and perform preoperative endoscopic resection (ER) for neoplastic lesions proximal to the primary lesion. The objective of this study was to determine the usefulness and safety of preoperative TCS and ER after SEMS placement in patients with obstructive colorectal cancer.

**Methods:**

From April 2016 to March 2022, we enrolled 100 patients with obstructive colorectal cancer who underwent SEMS placement, including 86 patients who underwent preoperative TCS after SEMS placement. Complications associated with preoperative TCS and ER after SEMS placement and the characteristics of the neoplastic lesions were assessed.

**Results:**

The success rate of SEMS placement as bridge-to-surgery was 98.0%; six patients had associated complications. Preoperative TCS was performed 8 (range: 1–30) days after SEMS placement. Four patients had synchronous advanced cancers. Nine non-advanced synchronous cancers, 116 adenomas, and 18 sessile-serrated lesions were treated by preoperative TCS and ER after SEMS placement. No procedure-related complications, namely stent migration, bleeding, and perforation were observed. Forty-five patients underwent follow-up TCS 1 year after surgery. Only one patient with submucosal invasive cancer required a second surgery.

**Conclusions:**

Preoperative TCS and ER after SEMS placement was performed with no complications. This approach allows preoperative evaluation of the entire colon and the treatment of precancerous lesions. (240 words)

## Introduction

Placement of colonic self-expandable metallic stents (SEMS) is widely performed in patients with obstructive colorectal cancer as a bridge-to-surgery (BTS). This procedure avoids emergency surgery by resolving obstruction to the passage of stool and obstructive enterocolitis. Colonic SEMS placement for BTS not only minimizes postoperative complications and the risk of colostomy compared with emergency surgery [1–3], but also provides similar surgical outcomes and long-term prognosis compared with non-obstructive colorectal cancer [4]. Another important problem with obstructive colorectal cancer is the possibility of synchronous colon cancers proximal to the primary lesion. Assessing patients for synchronous proximal colonic cancers is very important because this issue affects the operative strategy for the primary lesion. In this regard, an important advantage of colonic SEMS placement is that this approach permits total colonoscopy (TCS) after preparation with osmotic laxatives before surgical treatment of the primary lesion. Preoperative TCS after colonic SEMS placement enables the assessment of the presence or absence of synchronous proximal colon cancers. This approach also permits preoperative endoscopic resection (ER), if applicable, for neoplastic lesions proximal to the primary lesion by cold snare polypectomy (CSP), endoscopic mucosal resection (EMR), or endoscopic submucosal dissection (ESD). The preoperative pathological diagnoses of synchronous colonic neoplastic lesions is important when considering the operative strategy for the primary lesion. The objective of this study was to determine the usefulness and safety of preoperative TCS and ER after colonic SEMS placement in patients with obstructive colorectal cancer.

## Methods

From April 2016 to March 2022, 100 patients with obstructive colorectal cancer admitted to our hospital underwent colonic SEMS placement for BTS. The Niti-S colonic stent (Century Medical, Co. Ltd., Tokyo, Japan) or HANAROSTENT Naturfit colonic stent (Boston Scientific, Marlborough, MA, USA) were used. In all cases, plain abdominal X-rays were obtained 2 days after colonic SEMS placement to confirm sufficient expansion. Eighty-six patients underwent preoperative TCS after preparation with an osmotic laxative (MoviPrep®; Salix Pharmaceuticals, Morrisville, NC, USA). Forty-seven patients underwent ER, which comprised CSP, EMR, or ESD, after stent placement (Table 1). The ER method was chosen on the basis of the lesion size (CSP: < 9 mm, EMR: 10–20 mm, ESD: > 20 mm). Regarding TCS after colonic SEMS placement, the colonoscope was inserted and ER was performed as gently and carefully as possible using small-caliber colonoscopes (PCF-PQ260L or PCF-H290ZI; Olympus, Co. Ltd., Tokyo, Japan). Patients underwent postoperative TCS 1 year after surgery. The data were expressed as median (interquartile range), if applicable. The approval of the ethics committee review board in our hospital was obtained for this study.

**Table 1:** Locations of the colonic SEMS placed for obstructive colorectal cancers

Location	Number of cases
Cecum (n)	3
Ascending colon (n)	5
Transverse colon (n)	12
Descending colon (n)	14
Sigmoid colon (n)	41
Rectum, RS (n)	20
Rectum, Ra (n)	3
Total (n)	98

SEMS, self-expandable metallic stent; RS, rectosigmoid colon; Ra, rectum above the peritoneal reflection

## Results

**Fig. 1:**
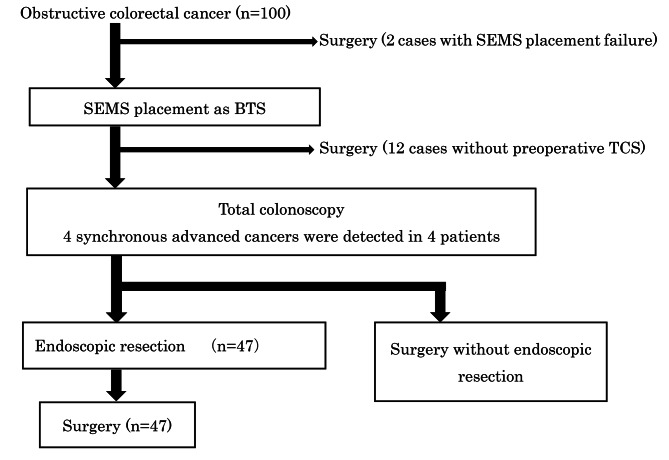
Clinical course of the patients with obstructive colorectal cancers CSP was performed for 102 lesions (38 patients) including 1 cancerous lesion EMR was performed for 38 lesions (19 patients) including 6 cancerous lesions (5patients) ESD was performed for 3 lesions (3 patients) including 2 cancerous lesions (2 patients) Nine synchronous non-advanced cancers were resected in eight patients SEMS: self-expandable metallic stent; BTS: bridge-to-surgery; TCS: total colonoscopy; CSP: cold snare polypectomy; EMR: endoscopic mucosal resection; ESD: endoscopic submucosal dissection

Of the 100 patients with obstructive colorectal cancer, colonic SEMS placement for BTS was performed successfully in 98 patients (Fig. 1). The success rate of colonic SEMS placement for BTS was 98.0%. The locations of the colonic SEMS are shown in Table 1. Five patients development complications (stenosis in one, perforation in four) associated with stent placement (Table 2). Preoperative TCS was performed 8 (range: 1–30) days after SEMS placement in 86 patients. Obstructive enteritis was observed in 12 patients, none of whom had abdominal symptoms, such as abdominal pain or vomiting, associated with preparation using osmotic laxatives (Table 3). Preoperative TCS after SEMS placement revealed that four patients had synchronous advanced cancers (Table 3). One hundred and two neoplastic lesions from 38 patients were treated by CSP (Table 4); 38 neoplastic lesions from 19 patients were treated by EMR (Table 5), and 3 neoplastic lesions from 3 patients were treated by ESD (Table 6). There were no complications, namely stent migration, bleeding, and perforation, associated with endoscopic insertion and treatment. Following preoperative TCS and ER after SEMS placement, 9 non-advanced synchronous cancers, 116 adenomas, and 18 sessile-serrated lesions were treated. Among 86 patients who underwent TCS before surgery, surgery was performed 25 (range: 8–47) days after colonic SEMS placement. Forty-five patients underwent follow-up TCS within 1 year after surgery. Regarding neoplastic lesions larger than 10 mm, seven lesions from six patients (cancer: two, adenoma: five) were detected (Table 7). Only one patient, with submucosal invasive cancer on the proximal side of the anastomosis, required a second surgery. The remaining six lesions were treated endoscopically by EMR or ESD.

**Table 2:** Complications associated with colonic SEMS placement among 98 patients with obstructive colorectal cancer

Adverse events after placement	Number of cases
Obstruction (n)	0
Stenosis (n)	1
Migration (n)	4
Perforation (n)	0
Total events rate (%)	5.1

SEMS, self-expandable metallic stent

**Table 3:** Characteristics of the patients who underwent total colonoscopy after colonic SEMS placement (n = 86)

Median days from stent placement to TCS (range)	8 (1–30)
Number of obstructive colitis cases detected during TCS (n)	12
Number of simultaneously detected synchronous advanced cancers (lesions/patients)	4 /4
Number of resected synchronous non-advanced cancers (lesions/patients)	9 /8
Number of total synchronous cancer (lesion/patient)	13 /11
Median days from stent placement to surgery (range)	25 (8–47)
Number of patients who underwent TCS within 1 year after surgery (n)	45

SEMS, self-expandable metallic stent; TCS, total colonoscopy

**Table 4:** Characteristics of 102 neoplastic lesions from 38 patients resected by CSP after colonic SEMS placement

Location	Carcinoma (invasion depth)	Adenoma	SSL	Total
Cecum (n)	0	6	4	10
Ascending colon (n)	1 (unclear)	18	2	21
Transverse colon (n)	0	23	4	26
Descending colon (n)	0	7	0	7
Sigmoid colon (n)	0	22	3	25
Rectum,RS (n)	0	2	1	3
Rectum, Ra (n)	0	5	0	5
Rectum, Rb (n)	0	3	2	5
Total (n)	1	86	15	102

CSP, cold snare polypectomy; SEMS, self-expandable metallic stent; SSL, sessile serrated lesion; RS, rectosigmoid colon; Ra, rectum above the peritoneal reflection; Rb, rectum below the peritoneal reflection

**Table 5:** Characteristics of 38 neoplastic lesions from 19 patients resected by EMR after colonic SEMS placement

Location	Carcinoma (invasion depth)	Adenoma	SSL	Total
Cecum (n)	0	1	1	2
Ascending colon (n)	1(M)	4	1	7
Transverse colon (n)	0	7	0	7
Descending (n)	0	6	1	7
Sigmoid colon (n)	5(M)	5	0	10
Rectum, RS (n)	0	2	0	2
Rectum, Ra (n)	0	1	0	1
Rectum, Rb (n)	0	3	0	3
Total (n)	6	29	3	38

EMR, endoscopic mucosal resection; SEMS, self-expandable metallic stent; SSL, sessile serrated lesion; RS, rectosigmoid colon; Ra, rectum above the peritoneal reflection; Rb, rectum below the peritoneal reflection

**Table 6:** Characteristics of 3 neoplastic lesions resected by ESD after colonic SEMS placement

Location	Cancer (invasion depth)	Adenoma
Transverse colon (n)	1 (in situ)	0
Rectum, Ra (n)	1 (submucosal, 2000 μm)	0
Rectum Rb (n)	0	1

ESD, endoscopic submucosal dissection; SEMS, self-expandable metallic stent; Ra, rectum above the peritoneal reflection; Rb, rectum below the peritoneal reflection

**Table 7:** Characteristics of neoplastic lesions larger than 10 mm detected in 45 patients who underwent follow-up colonoscopy within 1 year after surgery

Patient age	Stent location	Location of newly-detected tumors	Resection method	Pathological diagnosis (invasion depth)
81	T	Sigmoid colon	EMR	Adenoma
67	RS	Cecum	ESD	Adenoma
		Cecum	EMR	Adenoma
75	S	Descending colon	Surgery	Carcinoma (submucosal, 1500 μm)
65	T	Ascending colon	EMR	Carcinoma (M)
83	D	Ascending colon	EMR	Adenoma
77	A	Transverse colon	EMR	Adenoma

T, transverse colon; RS, rectosigmoid colon; S, sigmoid colon; D, descending colon; A, ascending colon; EMR, endoscopic mucosal resection; ESD, endoscopic submucosal dissection; M, mucosal

## Discussion

In obstructive colorectal cancer, the reported frequency of synchronous colon cancers is 9% [5]. Recently, preoperative TCS after colonic SEMS placement was associated with a non-negligible chance of detection of synchronous colon cancers proximal to the primary lesion [6, 7]. Additionally, preoperative TCS after colonic SEMS placement permits the assessment of the presence or absence of synchronous colon cancers as well as biopsy and/or ER [8]. This approach can provide information to determine the operative strategy for the primary lesion. In the present study, preoperative TCS and ER after colonic SEMS placement revealed synchronous colon cancers in 11 patients (12.7%). Both the expanded diameter of SEMS and the colonoscope caliber are key to success or failure with preoperative TCS after colonic SEMS placement [9]. In the present study, by gently and carefully inserting small-caliber colonoscopes, no complications were associated with preoperative TCS and ER after colonic SEMS placement.

It is important to note that colorectal cancer can be detected even in patients who undergo TCS between 6 months and several years before the diagnosis, namely post-colonoscopy colorectal cancer. Patients with a history of colorectal cancer surgery have a risk of developing post-colonoscopy colorectal cancer [10]. The Japanese Society for Cancer of the Colon and Rectum Guideline 2022 for the treatment of colorectal cancer recommends surveillance TCS 1 year after surgery [11]. The reported yield of colorectal cancer at surveillance colonoscopy 1 year after curative resection of colorectal primary cancer is 1.7% [12], and the yield could be even higher in patients with obstructive colorectal cancer who have not undergone preoperative evaluation of the entire colon. Thus, follow-up TCS is recommended within 6 months after surgery in patients who cannot undergo preoperative TCS owing to stenosis. In the present study, 87.4% of ER lesions were pathologically diagnosed as adenomas or adenocarcinomas. Considering that colonic adenoma may progress to adenocarcinoma, both adenocarcinomas and adenomas could be targets for preoperative ER. Therefore, it is important and effective to evaluate the entire colon, if possible, and to treat precancerous lesions before colorectal cancer surgery. In this study, only one patient with cancer detected by follow-up colonoscopy had to undergo a second surgery.

Being able to perform colonoscopy with a thin-diameter colonoscope through the SEMS provides very important advantages for the treatment of the patient, but colonic SEMS placement for the sole purpose of proximal colon observation or ER, but not for BTS, should be avoided. There were no complications associated with preoperative TCS and ER after SEMS placement in the present study. However, colonic SEMS placement is not recommended for patients with obstructive colorectal cancer without obstructive symptoms in accordance with the Japanese Society for Cancer of the Colon and Rectum Guideline 2022 for the treatment of colorectal cancer. This is because the procedure-related perforation rate is reported to be 1.6%, and the rate of the risk of deviation is reported to be 1.3% [11].

The limitations of this study are small sample size, for there is no data for long-term course but only short follow-up after surgery. More statistical analysis of those patients by longer follow-up is necessary.

In conclusion, preoperative TCS and ER after SEMS placement was performed without complications, in this study. This approach permits preoperative evaluation of the entire colon and the treatment of precancerous lesions, which can avoid unnecessary multiple surgeries in patients with obstructive colorectal cancer.

## Data Availability

The datasets generated during and analyzed during the current study are not publicly available due to due to restrictions for the availability of these data, which were used under the license for the current study but are available from the corresponding auther on reasonable request.
